# Effects of shallow natural gas well structures and associated roads on grassland songbird reproductive success in Alberta, Canada

**DOI:** 10.1371/journal.pone.0174243

**Published:** 2017-03-29

**Authors:** Jenny Yoo, Nicola Koper

**Affiliations:** Natural Resources Institute, University of Manitoba, Winnipeg, Manitoba, Canada; University of Sydney, AUSTRALIA

## Abstract

Grassland songbird populations across North America have experienced dramatic population declines due to habitat loss and degradation. In Canada, energy development continues to fragment and disturb prairie habitat, but effects of oil and gas development on reproductive success of songbirds in North American mixed-grass prairies remains largely unknown. From 2010–2012, in southeastern Alberta, Canada, we monitored 257 nests of two ground-nesting grassland songbird species, Savannah sparrow (*Passerculus sandwichensis*) and chestnut-collared longspur (*Calcarius ornatus*). Nest locations varied with proximity to and density of conventional shallow gas well structures and associated roads in forty-two 258-ha mixed-grass prairie sites. We estimated the probabilities of nest success and clutch size relative to gas well structures and roads. There was little effect of distance to or density of gas well structure on nest success; however, Savannah sparrow experienced lower nest success near roads. Clutch sizes were lower near gas well structures and cattle water sources. Minimizing habitat disturbance surrounding gas well structures, and reducing abundance of roads and trails, would help minimize impacts on reproductive success for some grassland songbirds.

## Introduction

The Great Plains region has experienced extensive habitat loss due to agricultural, urban and industrial development [1, 2]. The expansion of oil and gas infrastructure across the Plains has exacerbated habitat loss and degradation [3]. Currently, 81% of the over 200,000 gas wells in Canada are situated in Alberta [4], overlapping with Canada’s most extensive remaining tract of prairie grassland [5]. Potential effects of energy development include disturbance and changes to landscapes, habitat, natural ecosystem processes, wildlife populations, and species dynamics [6]. One consequence of habitat loss and land conversion has been a severe reduction in abundance of grassland songbirds [7], and there are increasing threats of further declines due to limited habitat protection [7–10]. Most prairies are now fragmented [2], thereby reducing the amount of available habitat for nesting birds, many of which are sensitive to habitat fragmentation [e.g. 11, 12].

Studies have shown both positive and negative effects of energy development on avian abundance or productivity, the magnitude of which may be dependent on the type of energy infrastructure, densities of energy development, habitat, and species [13]. Some studies have reported little evidence of negative effects of energy development on birds in boreal forests [14], although significant effects have been detected in other studies [15–17]. Energy development has been directly linked to population declines of greater sage-grouse *(Centrocercus urophasianus)* [18], while lesser prairie chickens *(Tympanuchus pallidicinctus)* selected nest locations farther from most energy related infrastructure [19, 20]. Surprisingly, nest success of passerines in shrubland ecosystems may be higher near compressor stations because nest predators avoid infrastructure noise [17].

The effects of shallow gas wells on grassland songbird reproductive success in Canada’s western prairies may be relatively small, as infrastructure is smaller, and associated levels of disturbance are lower, compared to many other types of energy infrastructure. For example, oil wells in the western prairies require permanent access roads, are noisy (56–81 dB(C) at 10 m; *n* = 64), and are visited by 1–15 vehicles per week [21]. In contrast, shallow gas wells average only 1.4 m in height, are silent [22], and disturbed areas (e.g., bare ground) associated with individual conventionally drilled shallow gas wells in this area are each only 1% of the footprint of oil wells. Further, shallow gas wells are typically accessed by trails rather than permanent gravel roads, and are visited once annually (usually) for maintenance [23]. Thus, it is reasonable to conclude that ecological effects of shallow gas wells may be much less than those caused by effects of oil wells. However, little research has been done to evaluate effects of shallow gas wells on grassland songbird productivity independently from effects of oil wells (cf. [24]).

Although the stature and disturbed area associated with shallow gas wells are small, shallow gas wells may still have some significant indirect effects on the surrounding ecosystem. Well infrastructure and associated roads may create edge effects, alter vegetation structure near wells, and contribute to the spread of exotic plant species [22, 25], which may lower nest or reproductive success of grassland songbirds [26]. Nest and reproductive success have a significant impact on avian population trends [27, 28], and thus one reason for declining grassland bird populations might be effects of energy development on their reproductive success.

Our study focused on the impacts of shallow gas wells on nesting grassland songbirds. Our objectives were to determine whether nest success or clutch size varied with shallow gas well structure density and with distance to wells, roads, and trails. We hypothesized that infrastructure presence might benefit the predator community by increasing edge effects, and that increased exotic plant species and altered vegetation structure near wells might limit food resources. Therefore, we predicted that nest success and clutch sizes would show a negative response to wells and associated roads and trails.

We also evaluated whether there were residual effects from well construction, as habitat removal, alteration, and/or restoration methods were used during well installment [3, 22]. Because exotic vegetation was briefly used for re-vegetation in the 1980s and early 1990s [29], and topsoil removal was common practice until the early 2000s (“full-build” construction methods; [30]), we predicted that the greatest ecological effects might be from intermediate-aged (15–30 years old) wells. We also predicted significant effects of new (< 15 years old) wells, as newer wells have had less time for vegetation to recover.

## Methods

### Ethics statement

All required animal care ethics were obtained for the research, including those from the University of Manitoba, Alberta Research permit (#49894), and Canadian Wildlife Service Scientific Take permit (11-MB/SK/AB_SC007, 12-AB_SC016). Privately owned land and land managed by Alberta Eastern Irrigation District were accessed, upon approval, for this research.

### Study sites and selection

Research was conducted in native mixed-grass prairies in southeastern Alberta, Canada, 2010–2012. Study sites were within a radius of 80 km of Brooks, Alberta, (approximately 50° 35'N, 111° 53'W, 760 m) and each site was 258-ha (1 sq. mile section; 2.580 km^2^) in area. We consider sites with zero wells to be controls, and treatment sites to be those with one or more gas wells. Potential sites were first selected using digital orthophoto satellite imagery to determine the presence of above ground gas well structures and to ensure that sites, and their surrounding landscape, were grasslands. Site suitability was confirmed by in-person site visits. Sites were at least 1 mile apart to ensure independence, surrounded by a grassland buffer at least 800 m wide.

In 2010, 37 sites were selected and surveyed. In 2011, we surveyed the same sites but eliminated one site because high rainfall made the site inaccessible. In the same year, we added four new sites, two of which were control sites (*n* = 40 sites). In 2012, 13 of the prior sampled sites were surveyed and one new site was added (*n*_2012_ = 14 sites). We surveyed 42 sites in total (*n*_total_ = 42 sites); despite the elimination of one site we were able to use site data; therefore, it was included (S1 Appendix).

**Fig 1:**
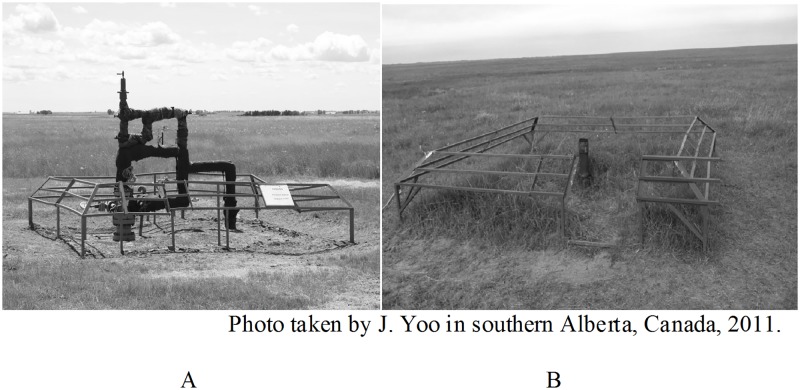
Pictures of a conventional active and non-producing gas well, both with cattle guard fencing. (A) Conventional shallow gas well, active and producing and (B) a non-producing well, both with cattle guard fences, southern Alberta, Canada.

Sites were dominated by native mixed-grass prairie vegetation, including blue grama (*Bouteloua gracilis*), needle-and-thread (*Hesperostipa comata*), western wheatgrass (*Pascopyrum smithii*), northern wheatgrass (*Agropyron dasystachyum*), prairie junegrass (*Koeleria macrantha*), and prairie sage (*Artemesia ludoviciana*). The most abundant exotic species, crested wheatgrass (*Agropyron cristatum*), was found in only 2% of our vegetation plots. Sites with at least an 800 m wide grassland buffer on all four sides were selected, to minimize risk of edge effects from surrounding croplands [12]. As is typical of mixed-grass prairies in this area, all our sites were grazed by cattle (*Bos taurus*). Livestock density and density of well pads were not correlated (*r* = -0.19, *p* = 0.28; [22]). Site topography was flat to gently rolling. Shallow-gas well densities ranged from 0–16 gas well pads / section (2.56 km^2^), which represented a maximum of 24 well heads / section due to commingling of wells on some well pads. Gas well densities were estimated based on GIS records and maps provided by Cenovus Energy Incorporated. Individual gas well pad footprints averaged 23.1 m^2^, and were at most 42.3 m^2^ (Fig 1A & 1B). Wells ranged in age from 1–44 years.

Roads provided access to sites within the study region, but rarely went directly to shallow gas wells. Roads included well-traveled gravel or dirt roads, and were on average 7 m in width. Trails directly linked gas wells to roads. Typically, vegetation on trails was abundant between tire tracks, or vegetation dominated the trail surface while small amounts of exposed bare ground was present in tire tracks; trails were on average 1.15 m in width. For all sites, a road to facilitate access bordered at least one edge of the 1x1 mile section, and multiple trails either bordered or were found within the site. On average, roads covered slightly more area within study sites (0.005%, SD = 0.004) than did trails (0.002%, SD = 0.0008), whereas gas well pad footprints covered less area than roads and trails, at 0%–0.0003% of each site.

Cattle water sources (i.e. stock ponds, dugouts) were included in the analyses because they were present in or adjacent to approximately half the study site sections, and have potential to influence nesting birds and bird food resources [e.g. 31–33]. Cattle water sources were from 250 m^2^–12,560 m^2^ in size, and covered 0%–0.005% of each site.

Potential nest predators present in the area included the American badger (*Taxidea taxus*), coyote (Canis *latrans*), Richardson’s ground squirrel (*Urocitellus richardsonii*), white-tailed deer (*Odocoileus virginianus*), mule deer (*Odocoileus hemionus*), pronghorn (*Antilocapra americana*), elk (*Cervus canadensis*), gulls (Family *Laridae*), common raven (*Corvus corax*), American crow (*Corvus brachyrhynchos)*, western meadowlark (*Sturnella magna*), brown-headed cowbird (*Molothrus ater*), and garter snake (Family *Colubridae*) [34–37].

### Nest searching and monitoring

We conducted two nest searches per year at each site to find and monitor songbird nests from mid-May—early August 2010, 2011, and 2012. Within each site, we searched for nests within two non-overlapping 100 X 1000 m survey plots. At control sites, survey plots were located randomly. At treatment sites, survey plots encompassed at least one well to ensure that we sampled a range of distances to wells. Nests were located by flushing incubating females from nests using the rope-drag method [e.g. 38]. Nests found by rope dragging and incidentally were marked by a bamboo stake to the south and pin flag to the west, both 10 m from the nest, and were monitored every 2–4 days until fail or fledge. Nests known or assumed to have fledged at least one offspring were considered successful. To determine nest fate, we used signs and cues at the nest, and behavior of adults and fledglings (e.g., excrement in the nest cup with a crushed cup rim, auditory or visual confirmation of fledglings near the nest, adults feeding or alarm calling near the nest).

### Vegetation surveys

We measured vegetation structure (density and % cover of live grass, dead grass, forbs, bare ground, shrubs, lichen, and moss; and vegetation height, litter depth, and occurrence of crested wheatgrass) after nests were completed. Although vegetation may have grown since nest sites selection, we did not measure vegetation until after the nest succeeded or failed to minimize disturbance to active nests. Our research elsewhere in Canada’s mixed-grass prairies demonstrates that vegetation structure is highly correlated within years, when measured as much as 2–3 months apart during the growing season (r = 0.684–0.831) [39] and thus it is reasonable to assume that our vegetation measurements provide a suitable index of vegetation structure during nest construction. Nonetheless, our measures of vegetation structure are relative indices of structure, rather than accurate measurements of nest site selection conditions. Vegetation was measured within 1x1-m quadrats centered on each nest. To measure vegetation density, we placed a Wien’s pole (6.3-mm diameter dowel marked in 10-cm intervals) vertically in the middle of the quadrat and at the four cardinal points counting the number of live, dead and invasive species stems or leaves touching the pole [40]. Vegetation height and litter depth were measured with a meter stick, and cover was visually estimated within each quadrant of the quadrat. Vegetation structure was also measured at two random locations within 50 m of each nest, to represent available but unused habitat (hereafter, “available habitat”).

### Preliminary analyses

Preliminary analyses were conducted to determine distributions of residuals and to determine which random and “nuisance” fixed variables should be included in final models. To assess the fit of residual distributions (normal, Poisson, negative binomial), we used the deviance/degrees of freedom ratio and diagnostic plots including box plots and/or Q-Q plots. We used a binomial distribution (success/fail) for nest success analyses.

To evaluate if nuisance or random variables should be included in our models, we compared the fit of models with and without random or repeated measure variables (site, nest), and the fixed-effects variables year and Julian day. For Generalized Estimating Equations (GEE) we selected the best model using the quasi-likelihood under the independence model information criterion (QIC) [S2 Table], and for logistic exposure models and generalized linear mixed-effects models we used Akaike’s Information Criterion modified for small sample sizes (AIC_*c*_) [S1 Table] [41]. Models with the lowest QIC or AIC units were selected as the best model, as our objective for these analyses was simply to ensure that influential variables were not ignored. However, the information theoretic approach was used only to ensure that we accounted for variability resulting from nuisance or random variables; for all subsequent analyses, we used a null-hypothesis significance-testing (NHST) approach, as we made specific predictions about the effects of individual variables based on our hypotheses [42]. When both “site” and “nest” were included as random effects in preliminary GLMM models, they failed to converge on several occasions. In these cases, the random effect “nest” parameter was estimated to be at or close to zero, suggesting it explained little to no variance in the data and thus was unnecessary. Therefore, we removed it from subsequent models. For all other analyses, a frequentist approach [42]. SAS 9.3 (SAS Institute Inc. 2008) was used to conduct both preliminary and primary analyses.

### Primary statistical analyses

We had sufficient data to evaluate effects of infrastructure on chestnut-collared longspur (*Calcarius ornatus*, *n* = 137) and Savannah sparrow (*Passerculus sandwichensis*, *n* = 120) nests in both preliminary and primary analyses. All chestnut-collared longspur and Savannah sparrow nests were included in our analyses, except for five weather-related failures (flooded nests), three abandonments caused by the research team, and ten nests visited infrequently due to access issues or undiscoverable after initial visit. When determining clutch size, parasitized nests were excluded from nest success analyses since parasitism may decrease clutch sizes. We were unable to evaluate effects of wells on parasitism since only two nests were parasitized during the three-year study. An alpha value of 0.1 was used to evaluate statistical significance to reduce the likelihood of making Type II errors (concluding there is no effect when there is an effect) [43].

#### Nest site selection and nest success

General Linear Mixed Models (GLMMs; PROC GLIMMIX) were used to determine whether vegetation density or structure differed at nests compared to available habitat (independent variable was nest/available habitat), as vegetation structure may influence nest site selection and nest success of grassland songbirds [25, 44, 45]. Analyses were conducted separately for chestnut-collared longspur and Savannah sparrow nests. Response variables included vegetation density (live, dead, or crested wheatgrass) and structure (live grass cover, dead grass cover, forb cover, occurrence of bare ground cover, shrub cover, lichen cover, moss cover, shrub cover, height, litter depth, or crested wheatgrass cover) variables. Cover of crested wheatgrass was low (0.38%) [46], but was included in analyses as it was once used as a restoration method after well construction [30] and is known to reduce reproductive success for chestnut-collared longspurs [26]. Site was included as a random variable in all models as our preliminary analyses indicated that it improved model fit (S1 Table).

The logistic exposure method [47] (NLMIXED) was used to determine whether vegetation structure influenced nest success. Analyses were conducted separately for each species. Site was included as a random variable because preliminary analyses indicated that it improved fit (S1 Table). Additionally, in all cases, models for Savannah sparrow included the fixed effects of year and Julian day, because they improved fit for this species, while models for chestnut-collared longspurs did not (S1 Table). For these analyses, the response variable was success/fail, while the independent vegetation structure variables differed slightly between species. Independent variables for chestnut-collared longspur models included live and dead grass density, live and dead grass cover, forb, lichen/moss, bare ground cover, litter depth, and vegetation height. Independent variables for Savannah sparrow models included year, Julian day, live and dead grass density and cover, litter depth, vegetation height, forb and bare ground cover.

#### Effects on nest success and clutch size

Using the logistic exposure method [47], three separate models were developed to determine effects on nest success. In all cases, analyses were conducted separately by species, site was included as a random variable, and the response variable was success (1) or fail (0) of each nest. The models were structured as follows. (i) To evaluate overall effects of infrastructure and associated linear features, we modeled effects of gas well density, and distances to wells, roads, trails, and dugouts on nest success. (ii) To determine whether the effects of gas well density varied with well age, we modelled effects of well density, age of well, and their interaction on nest success. (iii) To determine whether the effects of distance of nests to gas wells varied with well age, we modelled effects of distance to nearest gas well, age of well, and their interaction on nest success.

Generalized Estimating Equations (GEE) were used to determine effects on clutch size; site was included as the repeated measure in both chestnut-collared longspur and Savannah sparrow models (S2 Table). In addition, Julian day was included as a fixed-effect for chestnut-collared longspur models (S2 Table). We used the same models as those used for nest success (i, ii and iii, above), but with clutch size as the response variable. The clutch size distribution was normal for all models, as confirmed with diagnostic graphs. We also added a non-linear model which, additionally, included a polynomial term (well age * well age) to determine if there was a non-linear relationship between clutch size and independent variables.

## Results

### Nest site selection

**Fig 2:**
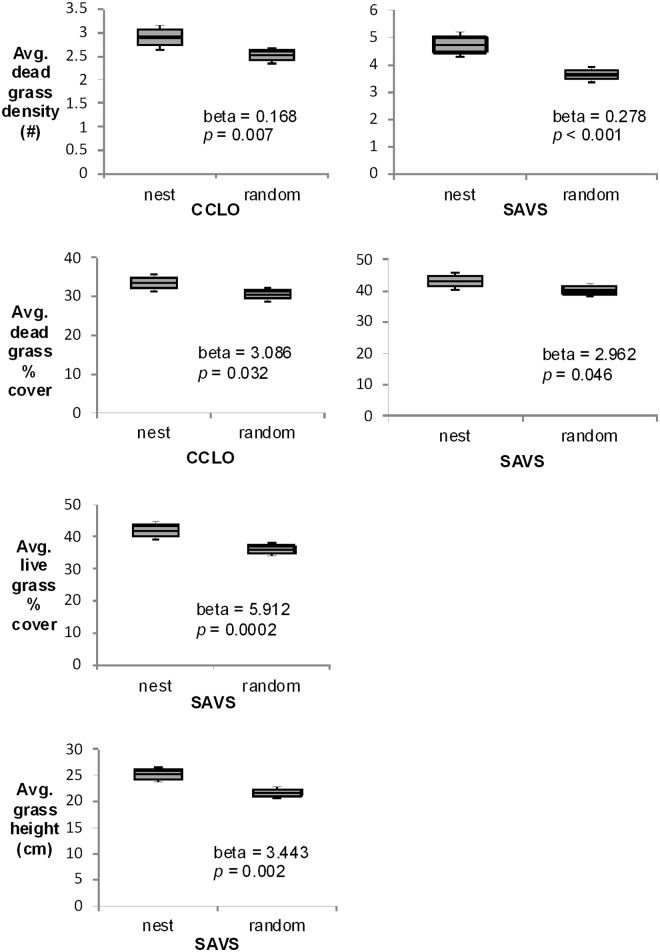
Vegetation structure at nests compared to available habitat. Vegetation structure at nests compared to available habitat for Savannah sparrow and chestnut-collard longspur nests in southern Alberta, Canada, 2010–2012. Only significant results (p < 0.1) are shown.

Savannah sparrows selected nest sites with 30–40% denser grass, 5–20% more grass cover, and 9–35% taller grass than available habitat (Fig 2). Similarly, chestnut-collared longspurs selected 6% denser grass and 8% more cover than available habitat; however, their nests had less dense grass and cover compared to Savannah sparrow nests (Fig 2). Savannah sparrow nests had less bare ground (β = -0.830, SE = 0.305, *p* = 0.010) and greater litter depth than available habitat (β = 0.209, SE = 0.081, *p* < 0.0001). Both chestnut-collared longspur (β = -4.507, SE = 1.712, *p* = 0.013) and Savannah sparrow (β = -1.313, SE = 0.252, *p* < 0.0001) nests had less lichen or moss than available habitat. The presence of crested wheatgrass (*p >* 0.308) and shrub cover (*p >* 0.130) did not vary between nests and available habitat, although occurrence of these elements was rare and thus our power to detect variability in these variables was low.

### Effects on nest success

**Fig 3:**
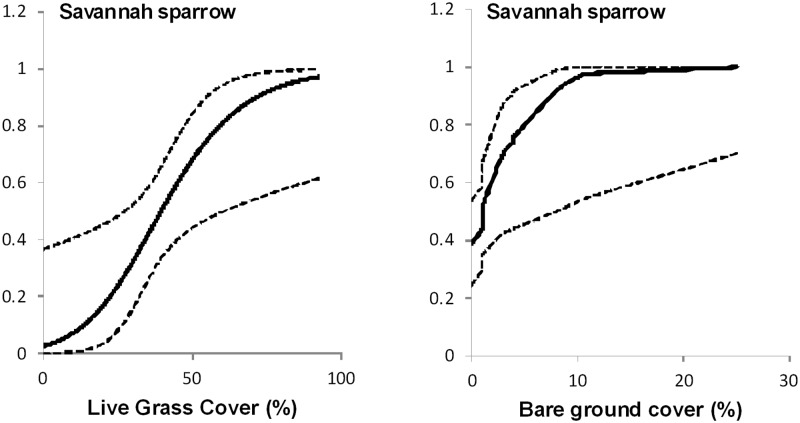
Effects of vegetation structure on nest success of Savannah sparrows. Effects of vegetation structure on nest success of Savannah sparrow in southern Alberta, Canada, 2010–2012. Only significant results (p < 0.1) are shown.

Savannah sparrow nest success was higher at nests with greater live grass cover (β = 0.0639, SE = 0.0314, *p* = 0.050) and more bare ground (β = 0.3519, SE = 0.1840, *p* = 0.064 (Fig 3). There was no influence of nest vegetation structure on success of chestnut-collared longspur (*p* > 0.202) nests.

There was no effect of distance to gas well structure on nest success of either species (*p >* 0.140) and this effect did not vary with well age (*p >* 0.134). Nest success was also independent of well density for both species (*p >* 0.886).

**Fig 4:**
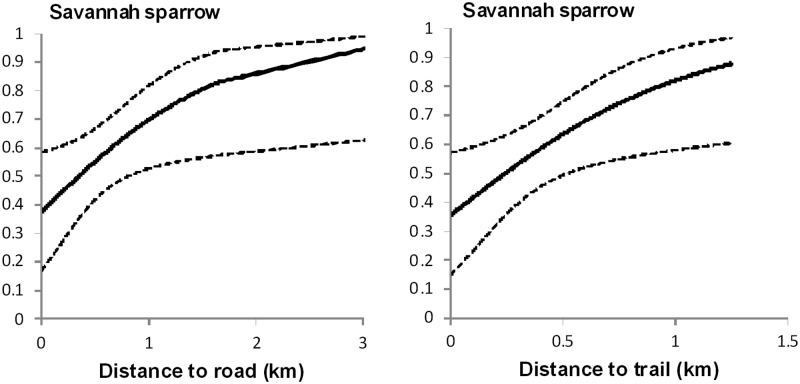
Effects of shallow natural gas wells, roads, and trails on nest success of Savannah sparrows. Effects of shallow natural gas wells, roads, and trials on nest success of Savannah sparrow in southern Alberta, Canada, 2010–2012. Only significant results (p < 0.1) are shown.

Savannah sparrow nest success increased further from roads (β = 1.016, SE = 0.548, *p* = 0.067) and trails (β = 1.673, SE = 0.864, *p* = 0.056) (Fig 4). Nest success was independent of distance to cattle water source (*p >* 0.249). There was no effect of roads and trails on nest success of chestnut-collared longspurs (*p* > 0.200).

### Effects on clutch size

**Fig 5:**
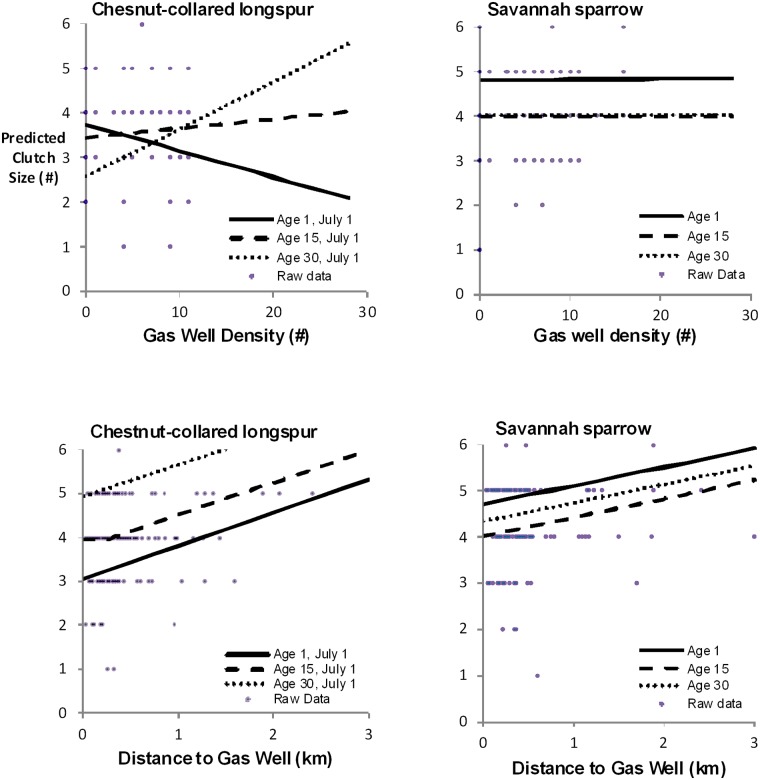
Effects of shallow natural gas wells, roads, and trails on clutch size. Effects of shallow natural gas wells, roads, and trials on clutch size of Savannah sparrow and chestnut-collared longspurs in southern Alberta, Canada, 2010–2012. Only significant results (*p* < 0.1) are shown.

On average, Savannah sparrow and chestnut-collared longspur clutch sizes increased as distance to gas wells increased, and there was a slight non-linear effect of well age for both species (Table 1, Fig 5). Savannah sparrow clutch sizes were greater by one egg near new wells (<15 years old) compared to wells of intermediate ages (15–30 years old) (Fig 5). Savannah sparrow clutch sizes were independent of gas well density, but there was a slight non-linear effect of well age (Table 1, Fig 5). Chestnut-collared longspur clutch sizes declined by two eggs in sites with high densities of new wells(< 15 years), but conversely, clutch sizes were predicted to increase by one to two eggs in sites with high densities of older wells (>30 years) (Fig 5).

**Table 1:** The effects of shallow gas well density, distance to wells, and well age on clutch sizes of Chestnut-Collared Longspurs (CCLO) and Savannah Sparrows (SAVS) in southern Alberta, 2010–2012.

		Density					Distance				
Species		Julian Day	Gas well density	Well Age	Gas well density * Well Age	Well Age * Well Age	Julian Day	Gas well distance	Well Age	Gas well distance * Well Age	Well Age * Well Age
**CCLO**	β	-0.0052	-0.0648	0.0146	0.0057	-0.0013	-0.0052	0.7725	0.079	-0.0319	-0.0014
(*n*_*interval*_ = *171*)	SE	0.0030	0.0290	0.0269	0.0016	0.0008	0.0030	0.2863	0.0295	0.0321	0.0007
	LCL	-0.0101	-0.1126	-0.0296	0.0031	-0.0026	-0.0101	0.3016	0.0304	-0.0847	-0.0025
	UCL	-0.0002	-0.0171	0.0587	0.0083	-0.0001	-0.0003	1.2434	0.1276	0.0208	-0.0003
	*p-value*	0.0861	0.0256	0.5876	0.0003	0.0797	0.0809	0.007	0.0075	0.3194	0.0314
**SAVS**	β	n/a	0.0006	-0.0928	0.0007	0.0021	n/a	0.4761	-0.0968	-0.0275	0.0027
(*n*_*interval*_ = 132)	SE	n/a	0.0231	0.0387	0.001	0.001	n/a	0.2413	0.0371	0.0194	0.0008
	LCL	n/a	-0.0374	-0.1565	-0.0009	0.0005	n/a	0.0792	-0.1578	-0.0595	0.0013
	UCL	n/a	0.0386	-0.0292	0.0024	0.0038	n/a	0.8729	-0.0357	0.0044	0.004
	*p-value*	n/a	0.9794	0.0164	0.4642	0.029	n/a	0.0485	0.0091	0.1566	0.0011

* Interaction

There was no effect of road (*p* > 0.159) or trail (*p* > 0.162) on Savannah sparrow or chestnut-collared longspur clutch size. Clutch sizes were higher further from water sources for Savannah sparrows (β = 0.046, SE = 0.024, *p* = 0.052) and chestnut-collared longspurs (β = 0.191, SE = 0.111, *p* = 0.087).

## Discussion

Overall, there were few effects of conventional shallow gas wells on productivity of grassland songbirds. We found some evidence that the presence of newer well structures within the last 15 years resulted in smaller clutch sizes. This suggests that wells may influence songbird resources or time/energy budgets, demographic structure, or food availability. Given that neither distance to well nor well density influenced the nest success of either focal species, our results suggest that nest failure caused by the presence of shallow gas wells is small, nonexistent, or compensatory [48]. As we found no effect of distance to shallow gas wells on relative abundance of Savannah sparrows or chestnut-collared longspurs in point-counts conducted in the same study sites [39], it is unlikely these patterns resulted from individuals selecting nest sites far from infrastructure.

One indirect effect of wells on Savannah sparrow nest success may be caused by trails. This is surprising, given that trails were partially or completely vegetated, rarely visited [23], and are apparently unobtrusive. However, roads and trails covered more area at our sites than gas wells, and thus their impact might be greater. Further, their linear nature may lead to their use as travel corridors by some predators such as foxes or coyotes, or they may host predator species that prefer edges [49]. Because Savannah sparrow nest success was lower closer to trails and roads but we found no corresponding effect on clutch size, this suggests that effects of roads and trails may have been mediated either by altered predator communities, or by altered availability of nests for depredation (e.g., higher visibility or detectability near roads). Habitat edges created by roads can alter plant and animal communities [50, 51], which can lead to numerous ecological effects [51] including either higher [52] or lower [53] rates of nest depredations. The variable outcomes among species may be due to species-specific habitat selection [44, 45, 54–56], nest concealment, behavior [24, 57–59], or predation risk [60].

While studies have found that grassland songbirds tend to select tall, dense vegetation for nests, this does not always lead to higher nest success [45, 53, 61]. Our study had similar findings; while both species selected greater cover than available, this only led to greater nest success for Savannah sparrow. Dense vegetation preferred by Savannah sparrows may protect some nests from visual predators, but because most depredations of songbird nests is opportunistic [62] and the predator community is diverse [63], vegetation cover most likely plays a small part in overall protection to many grassland songbird nests.

Despite shorter and exotic vegetation near gas wells [22, 39], these changes did not affect nest success. This suggests that sufficient vegetation remains on the landscape to allow individuals to select sites with suitable cover and protection from predators [64, 65]. Other research has demonstrated that impacts of shallow gas wells on vegetation structure are driven by both residual effects of well construction activities and by cattle grazing near wells [22]. Because cattle grazed all our sites, inference to other sites should be restricted to those grazed by livestock; however, as this characterizes the majority of the Northern Great Plains [66], its implications are still extensive.

While clutch sizes were independent of distance to roads and trails, chestnut-collared longspur clutches which range from 3–5 eggs per nest [67], tended to be smaller near wells, particularly when close to newer wells. Further, clutch sizes decreased as density of new wells increased, but surprisingly, clutch sizes increased as density of old wells increased. One possible explanation for this trend is that food availability may be lower near newer wells. Elsewhere we have shown that litter depth is shorter near newer shallow gas wells, but this effect decreases as wells age, presumably because vegetation destroyed during well construction gradually regenerates over time [22]. Short vegetation and higher abundances of exotic grasses near wells [22, 39] may lead to lower availability of invertebrates and seeds [68–70], which are primary food sources for chestnut-collared longspurs during the breeding season [71–73]. Therefore, food availability may increase as residual effects of well construction decline over time. Other possible explanations for lower clutch sizes near newer wells include higher stress levels in disturbed sites [74, 75], or older or higher-quality birds selecting territories farther from wells and displacing subdominant individuals to territories closer to newer wells [76]. Further research is required to test these hypotheses.

Clutch sizes of Savannah sparrows can range from 2–6 eggs [67] and were found lowest near wells of intermediate ages, perhaps because the use of exotic vegetation to restore well sites following drilling activities during the late 1980s and early 1990s reduced habitat quality around wells [3, 28]. Newer methods of well construction, which involve drilling directly through topsoil and the vegetation layer rather than removing topsoil prior to drilling, disturb significantly less vegetation than traditional “full-build” drilling methods [3]. Savannah sparrows and chestnut-collared longspurs demonstrate diet niche separation in grasslands [77], perhaps explaining why their responses to well age differ. As clutch sizes may influence population trends [78, 79], understanding these mechanisms may help mitigate short-term negative effects of well construction and may contribute to the conservation of these declining species. Our results suggest that negative effects of wells may decline over time, as wells age.

While we detected relatively few effects of shallow gas wells on productivity of grassland songbirds, cumulative effects of wells on clutch sizes might contribute to negative trends of these declining populations [79, 80]. Effects of wells on songbird productivity may be mediated by predators, vegetation structure, energy budgets, prey availability, competition, or combinations of all of these, and may be caused by residual effects from well construction or ongoing effects of the permanent infrastructure associated with wells [21]. As we observed effects of both newer and older wells, it suggests effects of wells may change as they remain on the landscape and presumably, some can be long-term. Effects of wells on different species also vary. Further research is required to identify which causal mechanisms explain effects of shallow gas wells on productivity of grassland songbirds, and whether wells influence other reproductive parameters, such as fledgling survival. Nonetheless, negative effects of shallow gas wells on clutch size, and of roads and trails on Savannah sparrow nest success, could both be mitigated by using commingled wells and directional drilling. This approach would minimize requirements for new well pads, roads, or access trails on the landscape thereby reducing effects of edge habitat fragmentation.

## Supporting information

S1 Appendix Study sites in southeastern Alberta. (DOCX)

S1 File Raw data. (XLSX)

S1 Table Random and fixed-effects variable model selection using Akaike’s Information Criterion (AIC_*c*_) in generalized linear mixed-effects. (DOCX)

S2 Table Repeated measure and fixed-effects variable selection using quasi-likelihood under the independence model information criterion (QIC) in Generalized Estimating Equations. (DOCX)
