# Supplementary material for: Effects of shallow natural gas well structures and associated roads on grassland songbird reproductive success in Alberta, Canada
Source: PLoS One. 2017 Mar 29;12(3):e0174243. doi: 10.1371/journal.pone.0174243 (PMC5371322; doi:10.1371/journal.pone.0174243)
Supplement: S1 Appendix — (DOCX) [file pone.0174243.s001.docx]

# S1 Appendix

Study sites (1.6 km x 1.6 km) located in southeastern Alberta for nest data collection in May-August 2010, 2011, and 2012 with

corresponding gas well densities. Yes (Y), No (N).

| Study  Site  # | Surveyed in 2010 | Surveyed in 2011 | Surveyed in 2012 | *Well Pad Density (wells/section)* | Study  Site  # | Surveyed in 2010 | Surveyed in 2011 | Surveyed in 2012 | *Well Pad Density (wells/section)* |
| --- | --- | --- | --- | --- | --- | --- | --- | --- | --- |
| **1** | Y | Y | Y | *0* | **22** | Y | Y | N | *6* |
| **2** | N | Y | N | *0* | **23** | Y | Y | N | *7* |
| **3** | N | Y | Y | *0* | **24** | Y | Y | Y | *7* |
| **4** | Y | Y | N | *0* | **25** | Y | Y | N | *7* |
| **5** | N | Y | N | *0* | **26** | Y | Y | N | *7* |
| **6** | Y | Y | Y | *0* | **27** | Y | Y | Y | *8* |
| **7** | Y | Y | Y | *0* | **28** | Y | Y | N | *8* |
| **8** | N | N | Y | *1* | **29** | Y | Y | N | *9* |
| **9** | Y | Y | N | *1* | **30** | Y | Y | Y | *9* |
| **10** | Y | Y | N | *1* | **31** | Y | Y | N | *9* |
| **11** | Y | N | N | *3* | **32** | Y | Y | Y | *9* |
| **12** | Y | Y | Y | *3* | **33** | Y | Y | N | *9* |
| **13** | Y | Y | N | *3* | **34** | Y | Y | N | *10* |
| **14** | Y | Y | N | *4* | **35** | Y | Y | N | *10* |
| **15** | Y | Y | Y | *4* | **36** | Y | Y | N | *10* |
| **16** | Y | Y | N | *4* | **37** | Y | Y | N | *10* |
| **17** | Y | Y | N | *4* | **38** | Y | Y | N | *11* |
| **18** | Y | Y | Y | *4* | **39** | Y | Y | N | *11* |
| **19** | Y | Y | Y | *5* | **40** | Y | Y | N | *11* |
| **20** | Y | Y | Y | *5* | **41** | Y | Y | N | *15* |
| **21** | Y | Y | N | *6* | **42** | Y | Y | Y | *16* |
