# Supplementary material for: Effects of shallow natural gas well structures and associated roads on grassland songbird reproductive success in Alberta, Canada
Source: PLoS One. 2017 Mar 29;12(3):e0174243. doi: 10.1371/journal.pone.0174243 (PMC5371322; doi:10.1371/journal.pone.0174243)
Supplement: S1 Table — (DOCX) [file pone.0174243.s003.docx]

S1 Table

Random and fixed-effects variable model selection using Akaike’s Information Criterion

(AIC*_c_*) in Generalized Linear Mixed-Effects.

| Variables | Model Selection:  Generalized Linear Mixed-Effects | | | |
| --- | --- | --- | --- | --- |
|  | Nest Site Selection and Nest Success | | | |
|  | CCLO | | SAVS | |
|  | AIC | AIC*c* | AIC | AIC*c* |
| Null | 415.6 | 415.6 | 326.4 | 326.4 |
| Null + No Random Effect | 414.9 | 414.9 | 325.3 | 325.4 |
| Null + No Random Effect + Year | 416.4 | 416.5 | 327.0 | 327.0 |
| Null + No Random Effect + Julian day | 414.7 | 414.8 | 318.6 | 318.6 |
| Null + No Random Effect + Year + Julian day | 415.9 | 415.9 | 318.2 | 318.2 |
| Null + Nest | 415.6 | 415.6 | 326.4 | 326.4 |
| Null + Nest + Year | 389.7 | 389.7 | 307.6 | 307.6 |
| Null + Nest + Julian day | 387.7 | 387.7 | 299.0 | 299.0 |
| Null + Nest + Year + Julian day | 389.0 | 389.1 | 298.7 | 298.7 |
| **Null + Site** | **231.4** | **231.4** | 224.1 | 224.2 |
| Null + Site + Year | 233.4 | 233.4 | 224.5 | 224.5 |
| Null + Site + Julian day | 233.1 | 233.1 | 222.7 | 222.8 |
| **Null + Site + Year + Julian day** | 235.1 | 235.1 | **221.4** | **221.5** |

Nuisance or random variables include “Site”, “No Random Effect”, and “Nest”.

Fixed-effects variables include “Year” and “Julian Date”.
