# Supplementary material for: Effects of shallow natural gas well structures and associated roads on grassland songbird reproductive success in Alberta, Canada
Source: PLoS One. 2017 Mar 29;12(3):e0174243. doi: 10.1371/journal.pone.0174243 (PMC5371322; doi:10.1371/journal.pone.0174243)
Supplement: S2 Table — (DOCX) [file pone.0174243.s004.docx]

S2 Table

Repeated measure and fixed-effects variable selection using quasi-likelihood under the independence

model information criterion (QIC) in Generalized Estimating Equations.

| Variables | Model Selection: Generalized Estimating Equations | |
| --- | --- | --- |
|  | Clutch Size | |
|  | CCLO | SAVS |
|  | QIC | QIC |
| **Site** | 177.0 | **134.4** |
| Site + Year | 178.2 | 135.0 |
| **Site + Julian day** | **173.2** | 134.6 |
| Site + Year + Julian day | 174.2 | 135.2 |

Repeated measure variables include “Site”.

Fixed-effects variables include “Year” and “Julian day”.
